# A comprehensive mixed-methods analysis of women’s cardiovascular health needs in Georgia, United States

**DOI:** 10.1186/s13293-025-00740-5

**Published:** 2025-08-11

**Authors:** Marlo Vernon, Brianna McIndoe, Michael J. Ryan, Amanda Behr, Daria Ilatovskaya, Ananya Chakraborty, Suma Yellamraju, Ara Idun, Jennifer Sullivan

**Affiliations:** 1 Department of Obstetrics and Gynecology, Georgia Prevention Institute, Medical College of Georgia, Augusta University, Augusta, GA USA; 2 Georgia Prevention Institute, Medical College of Georgia, Augusta University, Augusta, GA USA; 3 Department of Pharmacology, Physiology, and Neuroscience, School of Medicine Columbia, University of South Carolina, Columbia VA Health Care System, Columbia, SC USA; 4 Department of Medical Illustration, College of Allied Health Sciences, Augusta University, Augusta, GA USA; 5 Department of Physiology, Medical College of Georgia, Augusta University, Augusta, GA USA; 6 Medical College of Georgia, Augusta University, Augusta, GA USA; 7 Department of Physiology, Medical College of Georgia and The Graduate School, Augusta University, Augusta, GA USA

## Abstract

**Background and Aims:**

In the United States, cardiovascular disease (CVD) is the leading cause of death among both men and women; CVD and associated risk factors particularly affect women who live in rural areas. This mixed-methods analysis aims to explore cardiovascular health (CVH) risk factors and healthcare experiences among women in rural Georgia, to identify barriers to care, and to inform strategies for improving long-term health outcomes in rural communities.

**Methods:**

A convergent mixed methods design was utilized to evaluate CVH prevalence and associated environmental risk factors among women living in rural GA. Quantitative data from 159 Georgia counties were analyzed to compare rural and urban rates of CVD-related conditions and healthcare provider availability. Comparative analyses were performed between counties, urban and rural areas, and on sex differences. Concurrently, semi-structured interviews were conducted with 56 women and 11 healthcare providers to explore knowledge of blood pressure (BP) management, access to preventive services, and barriers to care. Qualitative and quantitative findings were analyzed separately and integrated during interpretation.

**Results:**

Rural counties have significantly higher prevalence of hypertension, obesity and stroke. General trends revealed higher rates of smoking, physical inactivity, and excessive alcohol consumption in rural counties compared to rates in urban counties of GA. Qualitative themes revealed affordability concerns, communication challenges between patients and providers, limited trust in telehealth, and the importance of delivering CVH education in community-based settings. Differences by age were also observed: younger women expressed less concern or awareness about CVH risks, while older women described greater engagement with care and health information. While the original aim included gaps in awareness and education, participants primarily described navigating systemic barriers across the care continuum.

**Conclusion:**

Rural women face individual, provider, and structural barriers to cardiovascular health and care. This unique study identifies chronic disease disparities and risk factors, with a higher disease burden observed in rural counties. Contributing factors may include limited resources for promoting healthy lifestyle choices, and reduced access to healthcare providers. Integrated findings underscore the need for sex- and gender- informed, age-specific, and community tailored strategies that address both health system access, and communication to improve CVH outcomes in underserved rural populations.

## Abstract (plain language summary)

This study looked at why women in rural Georgia, United States are at higher risk for high blood pressure and heart disease. Health data was gathered from all 159 counties in Georgia, US and women and health care providers were interviewed to understand their lived experiences with cardiovascular health.

## Highlights

Women in rural Georgia exhibited higher rates of hypertension, hyperlipidemia, and stroke compared to their urban counterparts.

Qualitative analysis identified key challenges such as affordability, limited access to care, communication barriers, and issues with technology and trust in healthcare providers. These factors contribute to disparities in managing cardiovascular health.

Younger women expressed less awareness or concern about cardiovascular health than older women, indicating a need for earlier, age-specific tailored education and outreach strategies.

## Background

In 2020, cardiovascular disease (CVD) resulted in approximately 19 million deaths globally, reflecting a ~ 20% increase since 2010 [1, 2]. In the United States, cardiovascular disease (CVD) is the leading cause of death among both men and women [3]. Georgia specifically experiences high rates of cardiovascular disease with 1 in 3 deaths, or more than 28,000 people, dying from CVD each year [4]. Among females in the US, the age-adjusted CVD death rate decreased from 382.9 deaths/100,000 population in 2010 to 342.0/100,000 in 2019; however, a 9.3% increase was observed to 2022 (376.9/100,000) [5]. This is also true for deaths due to CVD among women in the state of GA– 213.8/100,000 in 2010, decreasing to 192.4/100,000 in 2019, and then increasing to 200.7/100,000 in 2022 [6]. Despite the high prevalence of CVD in the United States, only 44% of women recognize it as their greatest health threat, according to the American Heart Association [7]. Nearly 45% of women aged 20 and older are living with some form of CVD (heart attack, stroke, heart failure, arrhythmia (irregular heartbeat), coronary artery disease, peripheral artery disease, aortic aneurysm, valve disease, and congenital heart defects), and less than half of pregnant women in the U.S. have good heart health [7]. Currently, around 47% of U.S. adults have hypertension. Of these cases, approximately 85% have no known cause, and among those receiving treatment, only 50%., achieve adequate control.

Hypertension a leading modifiable risk factor for both CVD and mortality [9], affects approximately 47% of adults in the US, with only half achieving adequate blood pressure (BP) control [8]. Although younger (ages 18–25) women are often perceived to have a lower risk of hypertension, conditions like high BP, preeclampsia, and gestational diabetes significantly increase their long-term risk of developing CVD [10]. In particular, women who experience gestational hypertension or preeclampsia during pregnancy are at increased risk of CVD or stroke 10 and 30 years after their pregnancy [11]. Additionally, the onset of menopause, marks a pivotal stage when cardiovascular risk factors, such as hypertension may accelerate, emphasizing the need for targeted health interventions during midlife [12].

Women in rural areas face elevated risks due to intersecting geographic, racial, and sex-related factors. Black women in the Southeastern U.S. are particularly vulnerable, experiencing higher rates of both CVD and maternal mortality [13–16]. Structural factors such as limited provider availability, economic instability, and social determinants of health (SDOH), including poor transportation access and healthcare mistrust, further exacerbate these disparities [7, 8, 17, 18]. Despite these alarming statistics, there remains a significant lack of research focusing specifically on women’s cardiovascular health. This gap in research impacts disparities by limiting the development of effective, sex-and gender-specific strategies to prevent and manage CVD.

In rural communities, negative social determinants of health (SDOH) are associated with increased hypertension rates and CVD risks. Research highlights that most cardiac events, including those linked to hypertension, are preventable with lifestyle changes such as increased physical activity, healthier diets, and better blood pressure management [8]. Economic instability, including poverty and unemployment, restricts access to healthcare, nutritious food, and medications critical for BP management [7]. However, rural women often lack the resources or opportunities to incorporate these changes into their lifestyle. Rural populations have less access to publicly available green spaces for physical activity, less access to grocery stores, and have higher rates of hypertension than those living in suburban or urban areas [2, 17]. Educational disparities can impact health literacy, complicating the understanding of hypertension risk factors and preventive care. Environmental barriers such as food deserts and insufficient recreational infrastructure contribute to poor diets and sedentary lifestyles, compounding the risk of hypertension [8]. Healthcare access in rural areas is also limited, where shortages of primary and specialty care providers are common, and long travel distances impede regular checkups and early intervention [17]. Black women, who often report experiences of structural racism or healthcare bias, report delays in seeking care due to mistrust of medical systems [13, 18]. This combination of challenges creates an environment where rural women, particularly Black women, face disproportionate risks of hypertension and CVD [15].

**Fig. 1:**
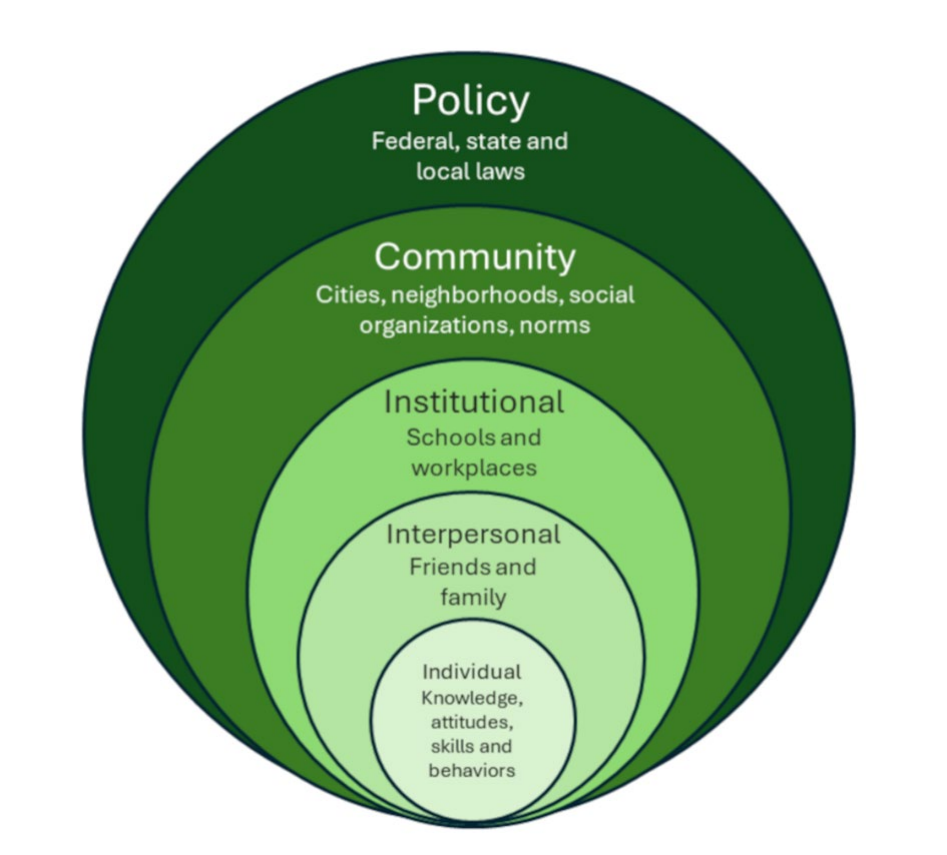
Socio-ecologic model of health (SEM). The model is a framework that considers many factors that influence health at multiple levels

This study presents a mixed methods approach to evaluating the CVH risks and healthcare experiences of women in rural Georgia, United States. While the primary focus is on women, population-level data were analyzed for both women and the general population. This broader approach was necessary because some key indicators were not available in a sex-disaggregated format. Presenting general population data where disaggregated data were unavailable allows for contextual understanding of county-level disparities that disproportionately affect women due to structural and socioeconomic vulnerabilities. Where available, sex-specific data were used to highlight risks unique to women.

Using a socio-ecologic model framework, we aimed to identify barriers to care, to inform future community-specific strategies to improve long-term CVH outcomes [19] (Fig. 1). We analyzed county-level CVD risk and outcomes data to highlight areas most in need of intervention, while concurrently conducting a qualitative analysis of rural women’s healthcare experiences and health care provider insights. This included knowledge of BP management and barriers to accessing preventive care. Central to this approach was the application of sex as a biological variable (SABV), recognizing that biological and social differences between men and women influence not only disease risk and outcomes, but also access to and experiences within the healthcare system. While the initial aim included understanding gaps in awareness, education, and access, the findings revealed that women’s narratives were more focused on their real-world experiences along the cardiovascular care continuum. These included how they sought information, accessed and utilized services, interacted with healthcare providers, and navigated the challenges of trust, communication, and health literacy in rural contexts. Therefore, this study reframes an individual’s cardiovascular health as a series of system-level interactions that shape women’s care experiences and outcomes.

## Methods

### Aim

The goal of this mixed methods study is to enhance understanding of hypertension risk factors and healthcare experiences among women in rural Georgia, in order to identify challenges and inform strategies for improving long-term cardiovascular health outcomes.

### Design

A congruent mixed methods design was used. Quantitative analyses of community health indicators were conducted concurrently with qualitative interviews to allow integration of findings. Quantitative results informed region-level trends, while qualitative interviews explored lived experiences of women and their healthcare providers, contextualizing the numerical patterns. Utilizing publicly available datasets (Table 1), a county level community health indicators analysis was conducted to compare rural vs. urban counties on prevalence of the cardiovascular health indicators and environmental risk factors. Secondly, we conducted a statewide qualitative approach, interviewing women and health care providers from across the state about their experiences, knowledge, beliefs, and attitudes around cardiovascular health and accessing care.

**Table 1:** County health indicators sources

Variable	Source	Time Frame
Access to Cardiologist	GA OASIS Data Analytics Center [23]	2021–2022
Access to PCP	GA OASIS Data Analytics Center [23]	2021–2022
Coronary Heart Disease (Women	CDC Interactive Atlas of Heart Disease and Stroke [24]	2019–2021
Coronary Heart Disease	CDC Interactive Atlas of Heart Disease and Stroke [24]	2019–2021
Diabetes Prevalence (Female	CDC Interactive Atlas of Heart Disease and Stroke [24]	2019–2021
Diabetes Prevalence (Male	CDC Interactive Atlas of Heart Disease and Stroke [24]	2019–2021
Excess Drinking Prevalence	County Health Rankings [25]	2023
HBP Prevalence (%) - Adults over 18	CDC Interactive Atlas of Heart Disease and Stroke [24]	2019–2021
HTN Prevalence (%)	GA All Payer Claims Database (APCD) [26]	2023
HTN Prevalence Women (%)	GA All Payer Claims Database (APCD) [26]	2023
Hyperlipidemia Prevalence (%)	GA All Payer Claims Database (APCD) [26]	2023
Hyperlipidemia Prevalence, Women (%)	GA All Payer Claims Database (APCD) [26]	2023
Obesity Prevalence	CDC Interactive Atlas of Heart Disease and Stroke [24]	2019–2021
Physical Inactivity Prevalence among all adults	County Health Rankings [25]	2023
Smoking Rates	County Health Rankings [25]	2023
Stroke Rate	CDC Interactive Atlas of Heart Disease and Stroke [24]	2019–2021
Stroke Rate (Women)	CDC Interactive Atlas of Heart Disease and Stroke [24]	2019–2021

### Community health indicators analysis

Data from 159 Georgia counties were evaluated to compare cardiovascular health (CVH) risk factors and healthcare access metrics between rural and urban counties. Counties were classified as rural or urban, with rural defined as having a population of less than 20,000, excluding military personnel. Urban/Rural classification followed the HRSA Rural Eligibility Tool definitions. Overall metrics for women and men and women only were evaluated as available. Key variables included the American Heart Association’s Life’s Simple 8 factors smoking status, dietary habits, physical activity, body mass index, blood pressure, total cholesterol levels, sleep and fasting glucose [20]. Additional CVD metrics, such as diabetes, hypertension, and hyperlipidemia prevalence rates, were also analyzed. High blood pressure was defined as blood pressure that is higher than the normal 120/80 mm Hg, while hypertension was defined as blood pressure that is consistently at or above 130/80 mm Hg, by the Centers for Disease Control and Prevention [21, 22]. Healthcare access was evaluated by provider type per 100,000 residents. The number of cardiologists and primary care physicians in rural versus urban counties was compared assessing healthcare access disparities. Data were sourced from multiple repositories, including the CDC Interactive Atlas of Heart Disease and Stroke, County Health Rankings, the Georgia All-Payer Claims Database (APCD), the Georgia Data Analytics Center, and the Georgia Department of Public Health’s Online Analytical Statistical Information System (OASIS) (Table 1). Independent samples t-tests were used to evaluate significant differences between rural and urban counties on the identified health metrics.

### Qualitative interviews

We conducted semi-structured interviews with 56 women and 11 licensed healthcare providers. Participants were recruited from five regional zones aligned with Georgia’s geographic and public health districts and the five satellite campuses of the Medical College of Georgia (MCG). It is important to examine the geology of regions when investigating rurality, as mountains vs. a coastal plain, for example, may impact technology access (cell service), travel times, and community resources differently. Inclusion criteria were women over the age of 18, who lived in a rural Georgia county (per HRSA criteria), or licensed healthcare providers who actively served women in these rural communities. This ensured that the sample was specific to the population of interest. Interviews focused on access, BP management, barriers, and recommendations for community-level interventions. Verbal consent was obtained, and interviews were recorded, transcribed, and de-identified.

A targeted recruitment strategy based on the county-level findings was used. Specifically, we used the results of the CVH indicator analysis to highlight rural counties with the highest prevalence, and prioritized participant recruitment from those areas (Dawson, Rabun, Oglethorpe, Putnam, Washington, Jefferson, McDuffie, Evans, Dodge and Sumter counties). Flyers and relevant information about the study were distributed to healthcare provider offices, public health departments, libraries, and community organizations. Efforts were made to recruit from locations frequently visited by the target population, such as libraries, farmers markets, gyms, health fairs, and healthcare facilities. These public spaces were chosen based on their location within rural communities. Some women also self-referred to the study by contacting the research team directly after seeing information on flyers.

Potential participants were screened to verify that they met the inclusion criteria. Women were asked to confirm their age, county of residence, and their willingness to participate in an interview about their rural healthcare experiences. Licensed healthcare providers were screened based on their work location and practice type to ensure they were relevant to rural women’s healthcare. Once screened and deemed eligible, participants were contacted to arrange an interview at a time that was convenient for them. Interviews were conducted based on the participants’ preference of either in-person or virtual (phone or computer) interviews to maximize participation.

Participants provided verbal consent after being read the informed consent document. This process ensured that participants fully understood their role in the study and the measures taken to protect their privacy. Interviews were semi-structured, following pre-established questions that allowed for both consistency across interviews and flexibility to explore participants’ unique experiences. The interview questions were created to gain information about participants’ healthcare experiences, their opinions on access to healthcare in rural areas, knowledge of women’s BP issues, challenges they experienced accessing healthcare and what support they believed are needed for healthy living in rural communities. Interviews were capped at 90 min to respect participants’ time, though most lasted around 30 min. All interviews were recorded with participants’ permission and then later transcribed.

After completing the interview, participants were emailed a link to a Qualtrics survey to collect demographic data. The survey asked participants to report their age range, county of residence, highest level of education completed, race/ethnicity, and technology usage in their household. These variables were collected to better understand the demographic profile of the participants and to identify how these characteristics may affect their healthcare experiences.

Each participant received a $25 gift card upon completing both the interview and demographic survey as a thank you for their time and participation. Gift cards were delivered via email using BHN Rewards.

To guarantee confidentiality, each participant was assigned a unique identification number. This number was used in place of personal information in all transcripts and records. Interview recordings were stored in a secure cloud folder, and access was restricted to authorized research personnel. Transcriptions were de-identified, removing any names or references that could link participants to their responses.

All interviews were recorded and transcribed. Two independent raters completed a multistep analysis for reviewing the transcripts, coding common themes separately for providers and participants. After individual thematic coding was completed, investigators discussed and agreed upon the main themes among the responses, and the interviews re-coded. A Kappa coefficient for agreement was estimated to determine inter-rater reliability using SPSS version 27 (IBM, 2020) for 35 selected quotes.

## Results

### Qualitative analysis

#### Demographics

A total of 56 women and 11 health care providers were interviewed (Tables 2 and 3. Demographics of Women and Health Care Providers).

**Table 2:** Demographics of qualitative respondents - women

	Women (*n* = 56)
**Age Group**	
18–25	7%
2635	21%
36–45	29%
45+	43%
**Sex**	
Female	100%
Male	
**Race/Ethnicity**	
Black/African American	23%
Hispanic/Latina	5%
White	75%
Native Hawaiian or Pacific Islander	2%
**Education**	
High School	21%
College	57%
Graduate School	21%
**Distance to Doctor’s Office**	
Within 10 miles	27%
10–20 miles	45%
> 30 miles	29%
**Heart Condition or High BP Diagnosis**	46%

**Table 3:** Demographics of qualitative respondents– health care providers

	Health Care Providers (*n* = 11)
**Age Group**	
36–45	27%
45+	73%
**Sex**	
Female	82%
Male	18%
**Race/Ethnicity**	
Black/African American	18%
Hispanic/Latina	
White	82%
Native Hawaiian or Pacific Islander	
**Education**	
College	27%
Graduate School	73%
**Total Number of Women Served Annually**	
50–100	18%
100–500	18%
500–1000	45%
> 1000	18%
**Total Women Served with Heart Conditions or High Blood Pressure Diagnosis**	
10%	18%
25%	18%
50%	45%
> 75%	18%

#### Inter-rater reliability

Independent rates identified 13 themes in their initial review, which was later distilled to four overarching themes. Cohen’s kappa was run to determine if there was agreement between the two raters on 35 selected quotes with the four distilled themes. There was good agreement between their classifications, k = 0.61, *p* < 0.001.

### Quantitative analysis

#### Risk factors

Rural counties exhibited significantly higher smoking rates in the overall population compared to urban counties, (17.9% vs. 22.5%, t(156) = 7.197, *p* < 0.001), physical inactivity prevalence (25.7% vs. 30.2%, t(73) = 6.10, *p* < 0.001), and obesity prevalence (36.7% vs. 38.9% t(56) = 2.77, *p* = 0.007). Conversely, urban counties demonstrated a significantly higher prevalence of excess drinking (overall population) (16.1% vs. 14.8%, t(152) = 2.68, *p* = 0.008).

### Theme 1: affordability and access to care

Quantitative data showed that rural counties had significantly fewer cardiologists and primary care providers per 100,000 residents (Table 4.). Notably, the distribution of healthcare providers was also unequal within both urban and rural categories. Among rural counties, 104 out of 120 (86.7%) lacked access to any cardiologist within their county; in contrast, 15.4% of urban counties did not have cardiologists within their borders. For primary care providers, while urban counties consistently had access, the density varied widely. Cobb, DeKalb, Fulton, and Gwinnett counties had access to over 200 primary care providers per 100,000 population, while most other counties—both urban and rural—had fewer than 100 primary care providers per 100,000 population.

**Table 4:** Average County health indicators by urban/rural classification

	Urban (*n* = 39)	Rural (*n* = 120)
County Population	219,277.4	20,645.1
County Population Female	112,971.9	10,402.6
Access to Cardiologist (rate per 100,000)	10.9	0.4
Access to PCP (rate per 100,000)	66.6	5.5**
Coronary Heart Disease Rate (rate per 100,000)	73.2	93.1**
Coronary Heart Disease-Women (rate per 100,000)	50.4	64.9**
Diabetes Prevalence Female	9.7%	8.6%*
Diabetes Prevalence Male	10.6%	9.6%*
Excess Drinking Prevalence	16.1%	14.8%
High Blood Pressure Prevalence	38.0%	43.6%**
Hyperlipidemia Prevalence	24.9%	31.3%**
Hyperlipidemia Prevalence Women	25.2%	32.6%**
Hypertension Prevalence	26.8%	35.7%**
Hypertension Prevalence Women	27.3%	36.0%**
Obesity Prevalence	36.7%	38.9%**
Physical Activity Prevalence	74.3%	69.8%
Physical Inactivity Prevalence	25.7%	30.2%**
Smoking Rates	17.9%	22.5%**
Stroke Rate (rate per 100,000)	45.6	47.1
Stroke Rate-Women (rate per 100,000)	43.4	44.7

** significant at *p* < 0.01

**significant at *p* < 0.001

P value results are from two sample t tests

#### Affordability of care and resources

In support of these findings, women shared stories of delayed or foregone care due to cost, as well as the logistical challenges of long-distance travel. Women frequently cited the cost of healthcare as a significant deterrent, particularly when it came to seeking specialist care. One woman who reported that she was in active heart failure, did not have insurance and would be unable to afford her medications without financial assistance programs that the hospital offered.

“My medications are over $1400 a month. So, I had to pay that for two months before they got me at the Good News Clinic in Gainesville and I got approved to get my medicine for free. They approved me because I am in heart failure, otherwise I wouldn’t have been able to afford it.” (ID 1114, White woman, 45+).

One participant stated that she was told to monitor her BP at home, but she was unable to afford the cost of the monitor. She stated that “if it is between a monitor and buying my kids groceries, then I am buying the groceries”. (ID 1066, Hispanic woman, 36–45)

#### Access to care

The dearth of specialty care providers in rural areas was consistently highlighted by participants. Appointments were often hard to get, and transportation access to arrive on time often presented a challenge for participants:

“Sometimes it takes a few months to get seen by a specialist, and I’ll usually end up driving 45 minutes to an hour.” (ID 1130, White woman, 26–35).

”It was easier to get a follow-up appointment with my doctor than it was to get an initial appointment.” (ID 1130, White woman, 26–35).

Limited public transportation options and the distance to medical facilities compounded these difficulties. Proximity to urban areas throughout the state, such as Athens or Atlanta, appeared to play a role in mitigating some of the challenges, with women who lived closer to cities reporting fewer barriers than those in more remote rural areas. However, this was a secondary observation, as the barriers related to affordability, transportation and communication were reported regardless of location. Despite these barriers, all participants expressed a willingness to support someone close to them in seeking care and reported that they would attend at least one specialist appointment if referred.

Community advocates and resources like health fairs were also highlighted as important venues to access health education, but also identified as an opportunity for expansion:

“Besides the health fairs, there are some advocates that will come to patients to discuss if they’re unable to come.” (ID 1133, Black woman, 26–35).

”Within the community, we have advocates and representatives…but it’s not like people on the forefront to explain it better.” (ID 1133, Black woman, 26–35).

”I wish… I kind of wish they had some more community activities. I mean, we have a few like Valley K Roads, stuff like that. But maybe something just for older people. It’s not necessarily that far, go out and do a mile walk or… I don’t know, do something” (1114, White woman, 45+).

To identify unique intervention sites for future heart health educational interventions, participants were also asked how often they went to a salon to have their hair or nails done. 35 of respondents reported going at least once every three months, supporting salons as a possible outreach and education site. 46 of the respondents also reported that the primary place they check their BP is at their doctor’s office, again highlighting an opportunity for diversifying access to quality BP monitors and health education.

### Theme 2: communication disconnects

Another important finding was the disconnect in communication between healthcare providers and patients. While providers reported always or often discussing BP and cardiovascular health during visits, many women stated that these topics were not addressed or not communicated in a way that they could remember or understand.

“Honestly, I’m a nurse practitioner, so I know what [BP] numbers mean, but most of the time, I’m just told, ‘Oh, it’s good.’ I have to specifically ask for the range.” (ID 1104, White woman, 26–35).

“I don’t feel like they spend enough time with them. And what’s bad is, unless you have somebody that is in for me either in the healthcare industry or has been through stuff they don’t even know the right questions to even ask about it. But I’m sure if somebody was not knowledgeable, and they were doing somebody’s BP, they would be telling them what the top number means and what the bottom number. And what normal is and what hypertension and what’s not” (ID 1114, White woman, 45+).

”I would rather get an explanation of what those numbers mean to me.” (ID 1105, White woman, 45+).

”I get a lot of numbers, and I would rather have a discussion about that than just getting the number” (ID 1105, White woman, 45+).

”So, I feel a lot of times they [doctors] don’t give them, give anybody enough information. Do you know stroke signs? Do you know heart attack signs? Concerned with if you’re feeling dizzy to check your BP.” (ID 1114, White woman, 45+).

*Women describing their desire for better understanding and health education*.

One healthcare provider also identified that they are not always the first source when health related information is sought out:

“The first person people ask for health information is someone they know in the community or Dr. Google.” (ID 1116, White woman, 26–35).

“Yes. I can always Google it if I have a question” (ID 1098, White woman, 45+).

Several women, particularly in younger age groups, described never discussing heart health unless they raised it themselves. In contrast, older women more often mentioned routine BP monitoring and greater awareness of CVD risks. In particular, CVH and signs and symptoms specific to women was rarely discussed as reported by women participants.

“I never go to the doctor unless I know I need to have surgery. So going in, like a routine visit or anything like that, I’m not one to do that. But when I was younger, my 20s and 30s when I had really good insurance, 40s, when I went in, that really wasn’t talked about when you’re younger” (ID 1114, White woman, 45+).

This underscores a generational communication gap—older women were more likely to receive BP education during clinical visits or seek information independently, whereas younger women reported both limited engagement and reduced perception of personal risk. These findings support the need for earlier, tailored interventions that address younger women’s unique perceptions and motivations regarding heart health.

### Theme 3: technology barriers and telehealth trust

Participants and healthcare providers both commented that some women in their counties lacked reliable internet access at home, limiting their ability to use telehealth services or online patient portals. One provider stated that many of his patients did not have internet access at home or “even a cell phone”, indicating a lack of reliable way for these women to access online healthcare (ID 1088, Provider, 45+). One participant shared,

“Well, being here in the rural county, you really don’t have too much wifi signal… so it’d be bad to get on there with a doctor trying to describe your problems and it messes up and you don’t know what the doctor diagnosed you with.” (ID 1098, White woman, 45+).

Furthermore, many women expressed hesitancy about telehealth, citing concerns that doctors cannot accurately diagnose them without an in-person appointment:

“Face-to-face conversation because when face-to-face, I get your understanding of it. You can’t hide it behind a phone.” (ID 1133, Black woman, 26–35).

Another participant shared:

“I feel like telehealth is a good tool for mental health services or general questions, but for physical illnesses, in-person is better.” (ID1130, White woman, 26–35).

### Theme 4: opportunities for Community-Based education

Significant differences were also observed in the prevalence of cardiovascular-related diseases between urban and rural counties, highlighting a community wide need for intervention. Overall, when compared with urban counties, rural counties had significantly greater prevalence of high blood pressure (43.6% vs. 38.0%, t(104) = 6.86, *p* < 0.001), hypertension in the general population (35.7% vs. 26.8%, t(105) = 11.38, *p* < 0.001) and among women (36.0% vs. 27.3%, t(157) = 8.1, *p* < 0.001); hyperlipidemia in the general population (31.3% vs. 24.9%, t(115) = 8.82, *p* < 0.001) and among women (32.6% vs. 25.2%, t(157) = 7.5, *p* < 0.001); and coronary heart disease prevalence per 100,000 adults (CHD) (93.1 vs. 73.2; t(73) = -3.875, *p* < 0.001) among the total population and also among women (64.9 vs. 50.4, t(65) = 4.1, *p* < 0.001). However, stroke rates showed no statistically significant differences between urban and rural counties for the overall population or for women. Despite gaps in care access, women were eager for more community-based education. Many noted that everyday community spaces—like nail salons, churches, grocery stores, and libraries—would be ideal locations. Among 56 participants, 46 reported checking BP only at the doctor’s office, while 35 said they visited a salon quarterly. These findings support potential future interventions that meet women where they are.

### Integrated findings overview

This study employed a mixed-methods approach to understand the intersection of cardiovascular health risks and healthcare experiences among women living in rural Georgia. The quantitative analysis highlighted a stark rural-urban divide: rural counties consistently exhibited higher prevalence of CVD-related conditions, particularly hypertension, hyperlipidemia, and obesity, and had fewer primary care providers and cardiologists per capita. These statistical disparities flagged systemic limitations in resource allocation and healthcare infrastructure.

The qualitative data added depth and human context to these findings. Through interviews with 56 women and 11 healthcare providers, we captured lived experiences that explained and personalized the numeric trends. Women described challenges accessing care, difficulties affording medications, and skepticism toward telehealth—all of which reflect the statistical underpinnings of provider shortages and poor outcomes. Quotes from participants illustrated how systemic issues manifest in daily life: for example, participants confirmed that gaps in broadband access and provider availability limited their ability to receive timely and effective care.

By layering qualitative themes—such as communication breakdowns, affordability barriers, and culturally preferred community spaces—on top of the epidemiologic trends, the study offers a holistic portrait of cardiovascular health inequities. These integrated insights support the development of more tailored interventions that address not only clinical access but also trust, literacy, and community engagement.

## Discussion

This study is grounded in the application of a sex- and gender-based lens (SBV), which is essential to identifying and addressing the unique ways that women experience cardiovascular risk, health system navigation, and care outcomes. Women’s experiences of cardiovascular health are influenced by biological factors (such as pregnancy-related hypertension and menopause) and gendered social roles that shape healthcare access, communication, and perceptions of risk. By focusing exclusively on women’s narratives and county-level data relevant to female populations, we aimed to uncover structural and cultural dynamics that may not be visible through general population data alone.

The findings of this study reinforces the critical and multifaceted challenges women in rural Georgia regarding their cardiovascular health. The findings of the county health indicators analysis underscore a pronounced disparity in the distribution of healthcare resources, with rural counties being disproportionately underserved. Disparities in provider availability have important implications for timely access to preventive care and specialist services. The results of the qualitative study reflect the quantitative health indicator highlighting the barriers rural women face in accessing and managing their cardiovascular health and their perceptions of care. Of note, women who had easier access to an urban city center, reported less difficulty with specialty care access, highlighting the importance of reviewing the region and geography of rurality. However, financial and transportation barriers persisted. By integrating state-level epidemiologic data with in-depth qualitative narratives, we offer a layered understanding of both the scope and lived experience of these disparities.

A striking disconnect was revealed between healthcare providers and their patients regarding communication. While providers reported discussing CVH topics such as BP, many women stated these discussions did not occur or were not conveyed in a way that resonated with them. This highlights a need for more patient-centered communication strategies—plain language, teach-back methods, take-home materials, and health education specific to women’s CVH risks. Underscoring the need for targeted sex- and gender-informed approach, educational interventions tailored to different age groups, emphasizing the importance of early prevention and regular monitoring of BP and other cardiovascular risk factors.

The study also revealed generational differences. Younger women expressed limited concern about heart health, often not seeing it as a priority. These differences in perception across age groups are consistent with the fact that older women have more chronic disease burden, greater CVH risk, and higher rates of stroke and heart attacks than younger women [22]. This suggests that early prevention methods are not reaching this group effectively. Tailored outreach, particularly in digital and social spaces frequented by younger women, may help foster earlier engagement in CVH practices.

In interviews, affordability, transportation, difficulty finding a doctor, and getting appointments were the most reported challenges mentioned by both patients and healthcare providers. Women have a greater prevalence of negative health outcomes in nearly all areas assessed; therefore, it is likely that women experience a greater negative impact on their health. The cost of healthcare was frequently cited as a deterrent, even for basic care, and transportation challenges further compounded access issues. These findings highlight the importance of addressing systemic barriers, providing transportation assistance programs or offering sliding scale payment options, to ensure that care is both accessible and affordable.

Technology access remains a double-edged sword. While telehealth may offer convenience, lack of broadband internet access emerged as a challenge. Even among those with access, many women expressed hesitancy about telehealth, feeling that in-person visits were essential for accurate diagnosis and treatment. Policymakers and healthcare systems should consider the digital divide, both in infrastructure and in building trust, to ensure telehealth does not inadvertently widen disparities.While telehealth holds promise for improving access, addressing technological barriers and building trust in virtual care will be vital to its success in rural communities.

The idea that more health education within the community would be beneficial was expressed by multiple women and healthcare workers. Many identified local community-based locations as ideal places for receiving health information. Libraries, grocery stores, nail salons, churches and pharmacies were all cited as places where women would feel comfortable learning more about their health. These findings emphasize the importance of meeting women where they are, both physically and culturally, may be more successful in increasing awareness, screening, and early prevention. Incorporating health education into these spaces could bridge some of the gaps in awareness and access, particularly for underserved populations.

Importantly, by centering women’s voices and incorporating a sex-specific lens, this study contributes to a sparse but growing body of literature that prioritizes rural women’s cardiovascular health. It also adds value to the mixed-methods field by showing how qualitative interviews can both validate and expand upon quantitative trends.

Ultimately our findings highlight the need of multi-faceted interventions, including improved education, expanded access to care and tailored communication strategies, to address the unique needs of rural women and their CVH: infrastructure and policy changes to increase provider availability and broadband access; communication training and tools for providers; and community-driven health promotion efforts that are sex- and age-tailored. These strategies must be aligned and resourced if we hope to reduce preventable CVH disparities among rural women.

This study has some limitations. First, while the qualitative sample included a diverse group of 56 women and 11 health care providers from rural Georgia counties, the findings may not capture the full spectrum of experiences, particularly for younger women and non-English speaking populations. However, the goal of qualitative research is not statistical generalizability but to provide in-depth insights into complex experiences, which this study achieves.

The recruitment process relied on participants who were either self-referred or recruited from public spaces. This approach may have introduced selection bias, as it excluded individuals who may not have frequented these locations or who were unaware of the study. There were also challenges with recruiting an equal sample of women in the 18–25-year age bracket.

We did not collect data on insurance status or employment, which could influence access to care and financial burden. Future studies should include these variables to better characterize socioeconomic influences. Lastly, while some sex- and age-specific patterns emerged through the interviews, we did not conduct formal subgroup analyses by demographic strata due to sample size. Further work is needed to disaggregate experiences more rigorously by age, race/ethnicity, and income.

## Conclusion

This study highlights the significant barriers rural women face in accessing cardiovascular care, including affordability, transportation and limited provider communication. By integrating population-level health indicators with in-depth personal narratives, we demonstrate how systemic gaps—limited provider availability, affordability, technology access, and communication disconnects—translate into real barriers for women.

The findings emphasize the value of combining sex- and gender- informed approaches, with community-based strategies to meet women where they are. Tailored communication, combined with a cultural awareness of generational differences attitudes toward heart health, broadband expansion, and outreach in trusted community spaces are needed to reduce the burden of CVD among rural women.

Ultimately, improving rural women’s cardiovascular health will require multi-level solutions that account for clinical, social, and cultural dimensions of care. This study offers a framework to guide future intervention development and policy efforts rooted in the voices of the women most affected.

## Data Availability

All quantitative data are available from publicly accessible websites and have been cited as such in the manuscript. Transcripts from the interviews are available from the corresponding author upon reasonable request.
